# Model selection and sample size adaptation: HYPAZ trial

**DOI:** 10.1186/1745-6215-12-S1-A5

**Published:** 2011-12-13

**Authors:** Simon Bond, Adrian Mander, Duncan Jodrell, Joseph Cheryian

**Affiliations:** 1 Cambridge Clinical Trials Unit, UK; 2 MRC Biostatistics Hub for Trials Methodology Research, UK; 3 CRUK Cambridge Research Institute, UK; 4 Cambridge University Hospitals NHS Foundation Trust, UK

## 

Pazopanib is a new cancer drug that works by limiting the growth of new blood vessels in tumours. About half of patients who take pazopanib develop high blood pressure (hypertension). This side effect can make patients have to reduce or stop their cancer treatment, and can cause other health problems.

Understanding how pazopanib causes high blood pressure will help us to advise doctors how to treat the high blood pressure effectively, so that patients can continue to take their cancer treatment safely. A proposed explanation is that hypertension is caused by endothelial dysfunction as a consequence of reduced nitric oxide (NO) bioavailability.

The aim of a study (HYPAZ) is to investigate the relationship between blood pressure and NO bioavailability in cancer patients treated with pazopanib, who develop hypertension. The predictor variable observed is the change in blood pressure from baseline. The response variable will be change in NO bioavailability measured through the change in forearm blood flow ratio (of infused versus control forearm) in response to intra-arterial acetylcholine infusion. These data will give a greater understanding to aspects of pazopanib’s mechanism and relationship to hypertension. The primary analysis for the study is to model and quantify the relationship between NO bioavailability and blood pressure.

In planning the study there is little *a priori* evidence to suggest the shape, nor to quantify the strength, of any relationship. Hence an adaptive design is proposed to assess the evidence for any relationship and judge possible sample size revisions at an interim analysis. To perform model selection at an interim analysis is a novel feature of this adaptive design. We consider some of the operating characteristics of the proposed design.

